# New Drugs Appliances, and Things Medical

**Published:** 1891-05-02

**Authors:** 


### NEW DRUGS, APPLIANCES, AND THINGS MEDICAL.

[All preparations, appliances, novelties, etc., of which a notice is desired, should be sent for The Editor, to care of The Manager, 140, Strand, London, W.C.]

### COCA WINE.

Coca wine is so much used now as a tonic and general restorative, that there is a great variety of brands in the market. These, like the unmedicated article, must be chosen with care to suit the digestive peculiarities of the patient. Messrs. Walters and Son, 33, Eastcheap, have forwarded for examination a case of their coca wine. On evaporation and extraction, the presence of cocoaine was easily demonstrated. It is stated that a wine glass-full represents the medicinal virtues of a dram of coca leaf. The wine is pleasant to the taste and undoubtedly restorative. We have tried it in cases of simple fatigue, exhaustion from overwork, and the weakness following acute febrile conditions with very favourable results. More especially do we think that it is useful in the case of overwork; it seems to act in a more prolonged way, and not to leave the patient in a worn condition as merely alcoholic stimulation does. Patients speak of this brand very favourably as regards taste and odour, and no objections are made on the score of its being "medicine-like."

### PURE CANE SUGAR.

Recently some considerable attention has been called to the fact that a very large trade is being done in "doctored" sugars. Mr. Cassall has pointed out how beetroot sugar is crystallised and coloured with analine dyes so as to simulate the genuine cane sugar coloured. Not content with imitating the colour, the beet sugar has been scented artificially in order to cover the characteristic disagreeable odour attached to this form ot saccharine material. The reason these sophistications are made is to enable the fraudulent dealer to make a profit out of the cheaper material which has been altered to appear like the best products of the cane. The objections to beet sugar are: (1) Its poor sweetening power, which is much below that of sugar from the cane. (2) Its inability to preserve fruit for long in jams, syrups, &c. It is noticed that when these preserves are made with beet sugar they quickly ferment and go bad, to the great disgust of the careful manufacturer or housewife. Pure cane sugar is a most powerful preservative of organic materials, but sugar from the beet seems to undergo the process of fermentation with considerable ease. (3) Its peculiar smell, which to many persons is very disagreeable. The smell of cane sugar on the other hand is but slight, and what little there is is pleasant. We have been led to make these preliminary remarks from the examination of a series of sugars which the above firm have sent to us for notice. They comprise varieties which come under the head of moist crystallized, refined cane, fine, and pure cane. They all answered to the tests for pure cane sugar only, and consequently can be highly recommended. Messrs. James Philip and Co., Fenchurch Buildings, Fenchurch Street, E.C., have started a novel idea; they put up the sugars in bags of 14 lbs. and upwards, and so labelled that one can tell at a glance whether the sugar is from Demerara, Trinidad, or Barbadoes. We were pleased to find among the series a bag of real old-fashioned brown sugar, with those delightful little crystalline lumps in it. It is a good many years since we have come across such really "sweet" sugars as these are. Where pure cane sugar is used we are of opinion that it will be found far less unwholesome for dyspeptics than sugar that contains "beet" in any form. Besides the sugar we received a tin of excellent "golden syrup;" this on analysis proved to belong to the same series of pure products.

